# Systematic Construction and Validation of a Metabolic Risk Model for Prognostic Prediction in Acute Myelogenous Leukemia

**DOI:** 10.3389/fonc.2020.00540

**Published:** 2020-04-21

**Authors:** Yun Wang, Fang Hu, Jin-yuan Li, Run-cong Nie, Si-liang Chen, Yan-yu Cai, Ling-ling Shu, De-jun Deng, Jing-bo Xu, Yang Liang

**Affiliations:** ^1^Sate key Laboratory of Oncology in South China, Sun Yat-sen University Cancer Center, Collaborative Innovation Center for Cancer Medicine, Guangzhou, China; ^2^Department of Hematologic Oncology, Sun Yat-sen University Cancer Center, Guangzhou, China; ^3^Department of Gastric Surgery, Sun Yat-sen University Cancer Center, Guangzhou, China; ^4^Department of VIP Region, Sun Yat-sen University Cancer Center, Guangzhou, China; ^5^Department of Oncology and Hematology, Shenzhen Luohu District Hospital of Traditional Chinese Medicine, Shenzhen, China; ^6^Department of Hematology, The Fifth Affiliated Hospital of Sun Yat-sen University, Zhuhai, China

**Keywords:** acute myelogenous leukemia, clinical prognostic model, metabolism, nomogram, gene set enrichment analysis

## Abstract

**Background:** Acute myelogenous leukemia (AML) is a heterogeneous disease with recurrent gene mutations and variations in disease-associated gene expression, which may be useful for prognostic prediction.

**Methods:** RNA matrix and clinical data of AML were downloaded from GEO, TCGA, and TARGET databases. Prognostic metabolic genes were identified by LASSO analysis to establish a metabolic model. Prognostic accuracy of the model was quantified by time-dependent receiver operating characteristic curves and the area under the curve (AUC). Survival analysis was performed by log-rank tests. Enriched pathways in different metabolic risk statuses were evaluated by gene set enrichment analyses (GSEA).

**Results:** We identified nine genes to construct a prognostic model of shorter survival in the high-risk vs. low-risk group. The prognostic model showed good predictive efficacy, with AUCs for 5-year overall survival of 0.78 (0.73–0.83), 0.76 (0.62–0.89), and 0.66 (0.57–0.75) in the training, adult external, and pediatric external cohorts, respectively. Multivariable analysis demonstrated that the metabolic signature had independent prognostic value with hazard ratios of 2.75 (2.06–3.66), 1.89 (1.09–3.29), and 1.96 (1.00–3.84) in the training, adult external, and pediatric external cohorts, respectively. Combining metabolic signatures and classic prognostic factors improved 5-year overall survival prediction compared to the prediction by classic prognostic factors (*p* < 0.05). GSEA revealed that most pathways were metabolism-related, indicating potential mechanisms.

**Conclusion:** We identified dysregulated metabolic features in AML and constructed a prognostic model to predict the survival of patients with AML.

## Introduction

Acute myelogenous leukemia (AML) is one of the most common types of adult acute leukemia (1) and shows striking heterogeneity, with recurrent gene mutations and variations in disease-associated gene expression (2). Although intensive chemotherapy and haematopoietic stem cell transplantation are the most common treatments, AML is fatal in approximately half of younger patients and ~80% of older patients because of primary refractoriness, treatment-related death, or palindromia (3). Risk stratification of leukemia is indispensable to ensure accurate treatment. Recent studies have emphasized the vital role of cytogenetics and molecular genetic analyses in hematological malignancies as a new layer of leukemia pathogenesis. Guidelines from the European leukemia network (ELN) in 2017 indicated that molecular abnormalities in genes such as *NPM1, FLT3-ITD, CEBPA, RUNX1, TP53*, and *ASXL1* combined with karyotype abnormalities can be used as an effective and comprehensive stratification system for the diagnosis and treatment of AML (4). However, even patients in the same layer of ELN categories show different prognoses. For example, ~50% of the AML patients with t (5, 6) (q22; q22) *RUNX1-RUNX1T1* have a favorable prognosis according to ELN risk stratification but show poor prognosis after intensive chemotherapy (7). Thus, additional factors for risk stratification should be identified and combined with analyses of cytogenetic and molecular abnormalities for more accurate AML prognostic stratification.

Metabolic reprogramming has recently been recognized as a vital and distinguishing feature of tumor cells (8, 9). The pathogenesis, chemoresistance, and palindromia of leukemia are also closely related to abnormal glucose metabolism, amino acid metabolism, and lipid metabolism. It has been reported that leukemia-initiating cells preferentially perform glycolysis (5) and take up amino acids, the catabolism of which is elevated in leukemia stem cells (10). A regulator of lipid metabolism, *TPD52*, was reported to be overexpressed and related to poor prognosis in patients with AML (11). By disrupting the tricarboxylic acid cycle and eradicating leukemia stem cells, the combination of B cell lymphoma 2 inhibitor (venetoclax) and demethylated drugs (azacytidine) was found to induce more durable remission than demethylated drugs alone in older patients with AML (12–14). The first drug targeting tumor energy metabolism approved by the US Food and Drug Administration, the isocitrate dehydrogenase 2 (IDH2) enzyme inhibitor enasidenib, also showed encouraging efficacy for treating *IDH2*-mutated relapsed or refractory AML (15). Metabolic processes have been shown to play important roles in the pathogenesis and progression of leukemia. However, a metabolic signature panel has not been explored to accurately stratify patients with AML to predict prognosis and for treatment management. In this study, we constructed a metabolic prognostic model from a Gene Expression Omnibus (GEO) dataset, which was further validated in two independent adult (The Cancer Genome Atlas Acute Myeloid Leukemia, TCGA-LAML) and pediatric (Therapeutically Available Research to Generate Effective and Treatments Acute Myeloid Leukemia, TARGET-AML) databases to explore an efficient metabolic signature for the more accurate stratification management of AML.

## Materials and Methods

### Datasets and Data Collection

The gene expression profiles of three AML cohorts were retrieved and downloaded from the corresponding datasets. Raw microarray data of GSE37642 (16) datasets were downloaded from the GEO database (http://www.ncbi.nlm.nih.gov/geo/) and normalized between different arrays. RNA-seq data from the TCGA-LAML dataset and TARGET-AML datasets were obtained from the UCSX Xena website (https://xenabrowser.net/datapages/). Transcripts per million normalized values were employed for further analysis. Detailed clinicopathological data including patient age, sex, leukocyte count, percentage of blast cells, French-American-British classification, genetic risk category, and survival information were download from the relevant item page on the UCSX Xena or GEO dataset website. The metabolic pathway-related gene sets of “c2.cp.kegg.v7.0.symbols” in gene set enrichment analysis (GSEA) were utilized as candidate metabolic gene lists. Genes were selected for further AML-related metabolic signature identification only when they were listed in all included cohorts.

### Metabolic Signature Construction and Validation

The GSE37642 dataset was used as the training cohort to construct the metabolic risk model. Least absolute shrinkage and selection operator (LASSO) Cox regression analysis was adopted to identify the optimal weighting coefficient of the prognostic metabolic genes. The metabolic model was built according to the penalized maximum likelihood estimator with 1,000-fold cross-validation. The 1-SE criteria were employed to determine the optimal values of the penalty parameter λ. TCGA-LAML and TARGET-AML datasets served as the adult and pediatric AML validation cohorts, respectively. The metabolic risk score was generated for each patient according to the unified formula determined in the training cohort. Patients were further grouped into high- and low-risk groups according to the optimal cut-off of the metabolic risk score determined by the survminer package.

### GSEA

GSEA v4.0.2 software (http://software.broadinstitute.org/gsea/login.jsp) was used to identify potential biological pathways comparing the high- and low metabolic-risk groups using the c2.cp.kegg.v7.0.symbols gene sets. A metabolic signature was generated for the GSE37642 dataset using metabolic pathway-related gene sets from c2.cp.kegg.v7.0.symbols. Only validation cohorts were included for enriched pathway analysis. A nominal *p* < 0.05 was considered statistically significant. Gene cloud biotechnology information (GCBI) and cytoscape 3.7.2 was used to explore the interactions between model-related metabolic proteins and other known related proteins.

### Statistical Analysis

Time-dependent receiver operating characteristic (ROC) curves were drawn to assess the predictive performance of the metabolic signature in the three cohorts. The area under the ROC curve (AUC) was calculated using the survival ROC package. The confidence interval was measured by the bootstrap method. Overall survival (OS) was defined as the primary outcome and calculated as the date of diagnosis or study entry to death from any cause. Kaplan–Meier curves were draw using the “survival” package and compared using the log-rank test. Clinical and genetic information were explored for prognostic performance via univariable- and multivariable Cox analyses. The χ^2^-test or the Fisher's exact test was performed to compare category variables. A nomogram was used to visualize and integrate the metabolic signature and classic independent risk factors, age, and genetic risk score for OS, the consistency of which was assessed by calibration. The AUC was used to evaluate and compare the prognostic value of the candidate factors. All statistical analyses were performed using R software (version 3.6.0) and SPSS version 24.0 software (SPSS, Inc., Chicago, IL, USA). A two-sided *p* < 0·05 was considered statistically significant.

## Results

### Patient Selection and Characteristics

Three AML cohorts including a total of 879 patients with available survival data were included in the analysis. Seven hundred and fifty-five candidate metabolic genes were retrieved from the Kyoto Encyclopedia of Genes and Genomes (KEGG) metabolic pathway-related gene sets. GSE37642 was utilized as a training cohort to estimate the prognostic metabolic model, and patients in the TCGA-LAML cohort and the TARGET-AML cohort served as external cohorts for metabolic model validation. The workflow of data collection has been shown in (Supplemental Figure 1). Patients from the GSE37642 and the TCGA-LAML cohorts were adults with AML with a median age of 57 (range: 18–85) years and 59 (range: 18–88) years, respectively, whereas those in the TARGET-AML datasets were pediatric patients with AML with a median age of 10 (range: 0–23) years. (Supplemental Table 1) shows the detailed patient characteristics of the three included cohorts.

### Metabolic Risk Score Construction

**Figure 1:**
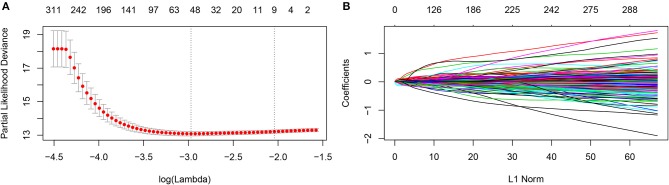
Construction of the metabolic model. **(A)** 1,000-fold cross-validation for variable selection in the LASSO regression via 1-SE criteria. **(B)** LASSO coefficients of metabolism-related genes. Each curve represents a metabolic gene.

The prognostic metabolic signature was trained using GSE37642 by the LASSO Cox regression. A penalized maximum likelihood estimator was performed with 1000 bootstrap replicates (Figure 1A). The optimal weighting coefficients were identified by the regularization parameter lambda via the 1-SE criteria (Figures 1A,B). Nine metabolic genes were selected for inclusion in the prognostic metabolic model. The formula for the metabolic model was as follows: metabolic risk score = 0·018 × *ALDH2* expression – 0·103 × *CYP2E1* expression + 0·078 × *DNMT3B* expression + 0·032 × *ENPP2* expression – 0·010 × *HAAO* expression – 0·039 × *ITPKA* expression – 0·007 × *PAFAH1B2* expression + 0·040 × *PHGDH* expression + 0·015 × *PSAT1* expression. The prognostic value of the selected metabolic genes was further assessed by the log-rank test after classification as high levels and low levels based on the corresponding optimal cut-off value in the training cohort (Supplemental Figure 2).

### Evaluation of Metabolic Risk Score

**Figure 2:**
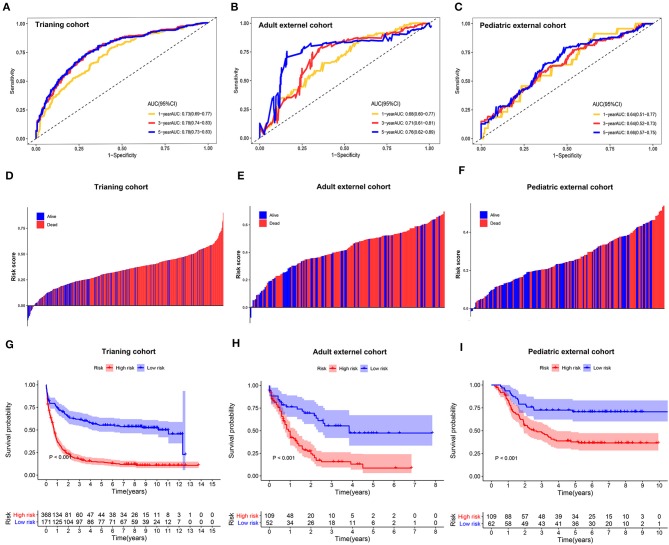
Time-dependent receiver operating characteristic (ROC) analysis, survival outcome analysis, and Kaplan–Meier analysis of the nine-gene signature in acute myelogenous leukemia (AML). **(A–C)** Time-dependent ROC analysis for 1-, 3-, and 5-year overall survival (OS) of prognostic model in training cohort, adult external cohort, and pediatric external cohort, respectively. **(D–F)** Distribution of risk metabolic score in survival outcome analysis for training cohort, adult external cohort, and pediatric external cohort. **(G–I)** Kaplan–Meier curve of the prognostic model in the training cohort, adult external cohort, and pediatric external cohort.

The sensitivity and specificity of the metabolic risk model were assessed through time-dependent ROC analysis. The AUCs for 1-, 3-, and 5-year OS were 0.73 [95% confidence interval (CI): 0.69–0.77], 0.78 [95% CI: 0.74–0.83], and 0.78 [95% CI: 0.73–0.83] in the training cohort, respectively (Figure 2A). The metabolic risk score was also calculated for each patient in the other two validation cohorts by the unified formula identified in the training cohort previously mentioned herein. The AUCs for 1-, 3-, and 5-year OS were 0.68 [95% CI: 0.60–0.77], 0.71 [95% CI: 0.61–0.81], and 0.76 [95% CI: 0.62–0.89] in the adult external cohort and 0.64 [95% CI: 0.51–0.77], 0.64 [95% CI: 0.52–0.73], and 0.66 [95% CI: 0.57–0.75] in the pediatric external cohort, respectively (Figures 2B,C).

We further analyzed the distribution of metabolic risk scores in patients with different survival statuses using a waterfall plot. Patients with lower metabolic risk scores generally had better survival outcomes than those with high risk scores (Figures 2D–F). Patients were then divided into high- and low-metabolic risk groups with the optimal cut-off determined in each cohort. Patients with a low metabolic risk had a significantly longer OS survival than those with a high metabolic risk level in the training cohort, adult external cohort, and pediatric external cohort (Figures 2G–I). The 5-year OS rates were 14.5% [95% CI: 10.8–18.22] vs. 55.2% [95% CI: 47.6–62.8] for the high- and low-risk groups in the training cohort, 8.7% [0.0–17.5] vs. 47.4% [30.9–63.9] for the high- and low-risk groups in the adult external cohort, and 70.7% [59.3–82.1] vs. 37.7% [28.3–47.1] for the high- and low- risk groups in the pediatric external cohort, respectively. Considering the potential effect of age on the metabolic gene expression, a sensitivity analysis was also performed in the younger patients (≤ 65 years) in both the training cohort and adult external cohort. After excluding the elder patients, those AML patients with a low metabolic risk still obtain a significantly survival advantage than those with a high metabolic risk in the population ≤ 65 years (Supplemental Figure 3).

### Univariate and Multivariate Analyses

**Figure 3:**
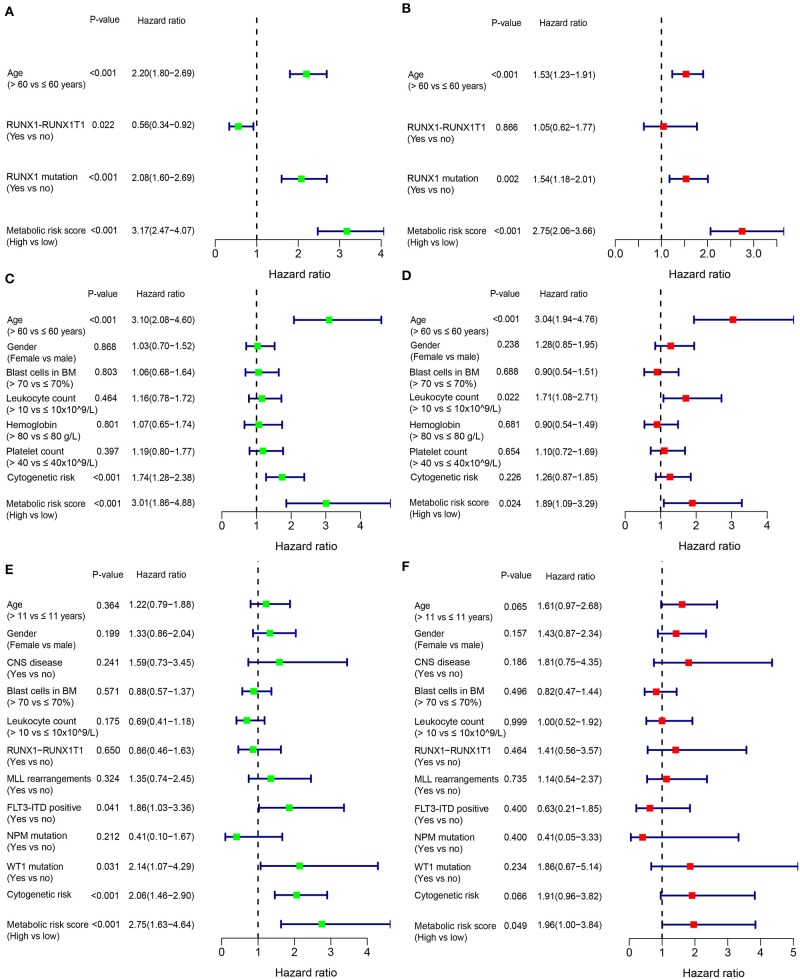
Forest plot of the univariate (left) and multivariate (right) Cox regression analysis in the training cohort **(A,B)**, adult external cohort **(C,D)**, and pediatric external cohort **(E,F)** for acute myelogenous leukemia (AML). BM, bone marrow; CNS, central nervous system.

In addition to the metabolic risk score, other prognostic values included age, *RUNX1-RUX1T1* fusion and *RUNX1* mutation in the training cohort (Figure 3A), age and cytogenetic risk category in the adult external cohort (Figure 3C), *FLT3-ITD, WT1* mutation and cytogenetic risk category in the pediatric external cohort (Figure 3E). After multivariable adjustments based on the other clinical factors, the metabolic signature remained as an independent prognostic indicator with a hazard ratio of 2.75 [95% CI: 2.06–3.66] in the training cohort (Figure 3B), 1.89 [95% CI: 1.09–3.29] in the adult external cohort (Figure 3D), and 1.96 [1.00–3.84] in the pediatric external cohort (Figure 3F).

### Clinicopathological Characteristics for Different Metabolic Risk Levels

**Figure 4:**
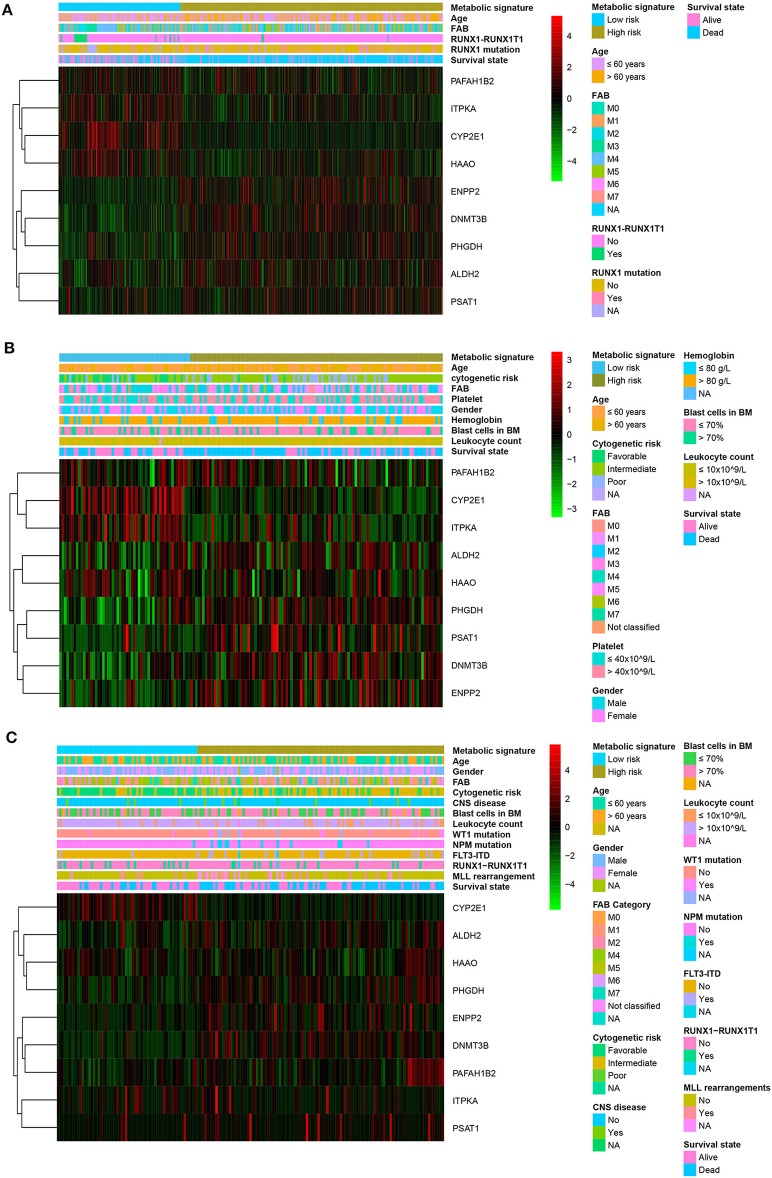
Heatmap of the nine-gene signature and clinicopathological characteristics at different metabolic risk levels for training cohort **(A)**, adult external cohort **(B)**, and pediatric external cohort **(C)**. Each column showing gene expression or clinicopathological state represents a sample and each row represents one characteristic or gene in the model. The expression levels of the nine genes are shown in different colors. Blue and yellow indicate low- and high-risk levels, respectively. BM, bone marrow; FAB, French–American–British classification; NA, not available; CNS, central nervous system.

Patients with high metabolic risk signatures were associated with an older age, lower percentage of *RUNX1_RUNX1T1*_fusion, higher percentage of *RUNX1* mutation, and higher platelet counts in the adult cohorts (including training or adult external cohort, Figures 4A,B and (Supplemental Table 1), as well as higher leukocyte counts and higher percentages of *FIL3-ITD* and blast cells in the bone marrow in the pediatric external cohort (Figure 4C and Supplemental Table 1). As expected, patients with higher metabolic risk levels had higher cytogenetic risk levels in all three cohorts (Figure 4 and Supplemental Table 1). Thirty-four paired samples from TARGET dataset were further used to analyse the metabolic risk difference between the disease at diagnosis and relapse, and results showed that the patients have a higher metabolic risk at the disease relapse compared with the status at the initial diagnosis (*P* = 0.022, Supplemental figure 4).

### GSEA

**Figure 5:**
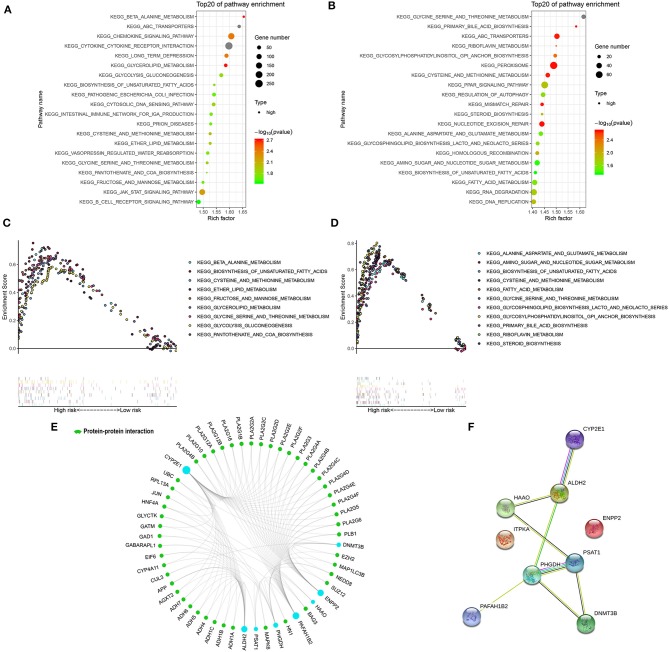
Significantly enriched KEGG pathways in adult external cohort and pediatric external cohort based on GSEA. **(A)** Top 20 representative KEGG pathways in high-risk patients in adult external cohort (*p* < 0·05). **(B)** Top 20 representative KEGG pathways in high-risk patients in pediatric external cohort (*p* < 0·05). **(C)** Representative metabolic pathways in high-risk patients in adult external cohort. **(D)** Representative metabolic pathways in high-risk patients in pediatric external cohort. **(E)** The protein-protein interactions between the metabolic model related proteins and the other proteins. The model related proteins are shown in blue circles, and the size of which is determined by the number of interacting proteins. ITPKA has no known interactions with other proteins. **(F)** The interactions of 9 metabolic proteins.

GSEA was performed in the two external cohorts to validate the metabolic-related pathway and explore other pathways enriched in different metabolic signature categories. Significantly enriched pathways were observed in the high metabolic risk group, most of which were metabolism-related pathways (Figures 5A–D). Among them, the biosynthesis of unsaturated fatty acids, fatty acid metabolism, and glycine, serine, and threonine metabolism were validated as enriched in the high-risk groups in both the adult and the pediatric external cohorts. Other metabolic signature-related pathways included the JAK-STAT signaling pathway, PPAR signaling pathway, mismatch repair regulation, regulation of autophagy, RNA degradation, and DNA replication (Figures 5A–D). Forty-eight proteins were explored from GCBI, which were interact with the 8 metabolic proteins in the model (Figure 5E), except for the ITPKA. In the protein-protein interaction (PPI) network, we found that the CYP2E1 and ENPP2 is interact with the proteins of phospholipase A2 family (PLA2G10, PLA2G12A, PLA2G12B, PLA2G16, PLA2G1B, PLA2G2A, PLA2G2C, PLA2G2D, PLA2G2E, PLA2G2F, PLA2G3, PLA2G4A, PLA2G4B, PLA2G4C, PLA2G4D, PLA2G4E, PLA2G4F, PLA2G5, and PLA2G6, Figure 5E). The interaction of nine metabolic proteins were summarized alone by STRING (Figure 5F).

### Comparisons of Prognostic Factors and Merged Risk Scores

**Figure 6:**
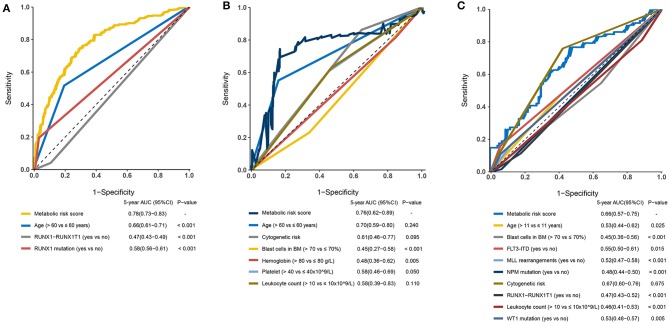
Time-dependent receiver operating characteristic (ROC) analysis of 5-year overall survival (OS) of metabolic risk model compared to other potential prognostic factors. **(A)** Time-dependent ROC analysis for 5-year OS of metabolic risk model was compared to age, *RUNX1-RUNX1T1*, and *RUNX1* mutation in the training cohort. **(B)** Time-dependent ROC analysis for 5-year OS of metabolic risk model was compared to age, cytogenetics risk, blast cells in bone marrow, hemoglobin, platelet, and leukocyte count in the adult external cohort. **(C)** Time-dependent ROC analysis for 5-year OS of metabolic risk model compared to age, blast cells in the bone marrow, cytogenetics risk, *RUNX1*-*RUNX1T1* fusion, *FLT3*-*ITD* status, *MLL*-rearrangement, *NPM* mutation, leukocyte count, and *WT1* mutation in the pediatric external cohort. BM, bone marrow.

The prognostic sensitivity and specificity of the metabolic signature were compared to those of other potential prognostic variables. The AUC for the 5-year OS showed a significantly higher metabolic risk score (0.78 [95% CI: 0.73–0.83]) compared to that with other variables such as age 0.66 [95% CI: 0.61–0.71], *RUNX1-RUX1T1* fusion (0.47 [95% CI: 0.43–0.49]), and *RUNX1* mutation (0.58 [95% CI: 0.56–0.61]) in the training cohort (all *p* < 0·001, Figure 6A). Additionally, in the adult external cohort, the metabolic risk model showed a numerically but no statistically larger AUC for the 5-year OS than the classic cytogenetic risk category (Figure 6B). In the pediatric external cohort, the 5-year AUC of the metabolic risk score was also equivalent to that of the cytogenetic risk category (Figure 6C).

**Figure 7:**
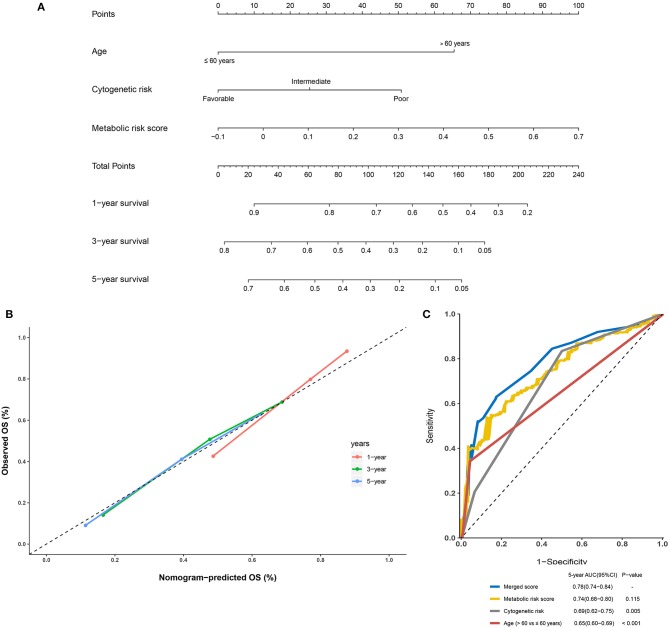
Building and validation of the nomogram to predict the overall survival of patients with acute myelogenous leukemia (AML) combining the adult external cohort and pediatric external cohort. **(A)** Nomogram plot was built based on age, cytogenetics risk, metabolic risk score, and total points combining the adult external cohort and pediatric external cohort. **(B)** Calibration plot of the nomogram. **(C)** Time-dependent receiver operating characteristic (ROC) curves of nomograms were compared based on a 5-year OS of AML.

Further, to generate a more accurate evaluation system, a nomogram was used to integrate the classic prognostic factors, age, and cytogenetic risk category and the present metabolic signature in the external cohorts (Figure 7A). The calibration plots showed that the nomogram could accurately predict the 1- and 3-year OS (Figure 7B). The AUC for the 5-year OS in the merged score was 0.78 [95% CI: 0.72–0.84], which was found to be significantly larger than that for the classic prognostic indicators including age (0.65 [95% CI: 0.60–0.69]) and cytogenetic risk category (0.69 [95% CI: 0.62–0.75]), indicating that adding the metabolic signature can increase the net benefit to predict OS compared to that with classic prognostic factors (Figure 7C).

## Discussion

Cancer cells rewire metabolic pathways to adapt to their increased nutritional demands for energy, reducing equivalents, and cellular biosynthesis (17). Leukemia cells vary the body's systemic physiology by impairing both insulin secretion and insulin sensitivity in the host to provide increased glucose to leukemia cells (18). Meanwhile, metabolism plays an important role not only in the development but also in the prognosis of leukemia (19, 20). However, prognostic models based on metabolic genes are lacking.

In the present study, a significant prognostic model based on nine metabolic genes was established in a training cohort and further verified in two independent external validation sets. The group that showed a high-risk score had poor prognosis, which was consistent across the three cohorts. The metabolic model showed a stably high prognostic value for AML, particularly for the survival of adult patients with AML. Moreover, the combination of classic prognostic factors including age and cytogenetic factors with our metabolic model improved the survival prediction compared to that with single classic risk factors, supporting the contention that the prognostic metabolic model can be utilized to supplement existing prognostic models.

In the present study, nine metabolism-related genes were identified and included in the prognostic model. The expression of *DNMT3B, ALDH2, ENPP2, PHGDH*, and *PSAT1* was negatively correlated with favorable outcomes, whereas the expression of *CYP2E1, HAAO, ITPKA*, and *PAFAH1B2* was positively correlated with favorable outcomes. Most of the nine genes in our model have been reported to be involved in cancer. *DNMT3B* has been shown to play a role in genic methylation and is involved in cysteine and methionine metabolism. High expression of *DNMT3B* is independently associated with adverse outcomes in older patients with CN-AML (6, 21), which is consistent with our results. Another study verified that the ectopic expression of DNMT3B can promote the development of gastrointestinal cancers via the *de novo* methylation and transcriptional silencing of the tumor suppressor genes in mice (22). The *ITPKA* gene also participates in inositol phosphate metabolism, and was found to be hypermethylated in patients with AML with a normal karyotype (23). *ENPP2*, which encodes the enzyme autotaxin, was found to be over-expressed in various cancers (24). A previous study reported that *FLT3-ITD* mutations in AML are closely associated with the high expression of *ENPP2* and may influence disease prognosis via the dysregulation of metabolism-related genes such as *ENPP2* (25–27). As the metabolism-associated gene with the highest weight in the model, *CYP2E* showed a positive association with the prognosis of patients with AML in our study. This enzyme plays a vital role in the production of reactive oxygen species and is involved in drug metabolism. The PPI network in our study also suggested the interaction between the phospholipase family and CYP2E1 and ENPP2. Arachidonic acid metabolism pathway and ether lipid metabolism pathway can be the potential interaction of the phospholipase family and the two metabolic genes (28, 29). Although the role of *CYP2E1* expression in the pathogenesis of AML remains unclear, polymorphisms in these gene have been shown to be associated with the risk of leukemia but not the risk of treatment-related leukemia (30–32). Decreased ALDH enzyme activity may lead to DNA damage due to the accumulation of aldehydes (33). In this study, *ALDH2* was an adverse prognostic factor in the model. A decrease in the ALDH2-GA or ALDH2-AA genotype was reported to accelerate the conversion of Fanconi anemia to MDS/AML (34). *PAFAH1B2*, which is involved in ether lipid metabolism, may also be broadly dysregulated in many types of cancer (35) and its over-expression at the transcriptional and at the protein levels in *MYC*-negative high-grade B-cell lymphomas is associated with good prognosis. Interestingly, we found different expression trends for *PAFAH1B2* between the childhood and adult leukemia metabolic profiles. This may be because of variations in the age-related regulation of the metabolism-related signature between adults and children. Other selected variables including *HAAO, PHGDH*, and *PSAT* were also found to predict the prognosis of AML. However, their mechanism with respect to AML remains unclear and requires further clarification.

As expected, the most significantly enriched pathways were metabolism-related in the GSEA, confirming the metabolic-related characteristics of the nine-gene signature. The high enrichment of metabolism-related and DNA repair-related pathways in the high-risk group in both independent validation cohorts indicates the potential benefit of targeting metabolism-related genes and PAPR inhibitor therapy for this population. However, the predictive value of the metabolic signature for these therapies should be further validated based on a large cohort in prospective trials.

We first focused on the role of metabolic genes in the prognosis of AML and constructed a prenotice significant model for AML stratification. However, several issues must be resolved. First, some clinical information such as the history of metabolic disorders and therapeutic information are lacking because this information was not available in public databases. Therefore, it is difficult to evaluate the association between metabolism and therapy and to avoid the inclusion of non-leukemia-related metabolic events. Moreover, validating our model in the real world is indispensable for extrapolating the established model to other AML populations, particularly childhood AML patients. Functional experiments are also needed to determine the mechanisms underlying the effects of the prognostic metabolic genes in AML. Finally, the diagnostic value of the metabolic risk score was not evaluated in this analysis, and need to be further explored in the perspective study.

## Conclusion

We established a prognostic metabolic model based on the metabolism-related genes in AML. The characteristic metabolic genes may reflect the disordered microenvironment of the bone marrow and may be used as potential biomarkers for AML prognosis. Validation of the model in the real world and functional experiments of the predictive metabolic genes are needed.

## Data Availability Statement

Publicly available datasets were analyzed in this study. This data can be found here: GSE37642, TCGA-LAML, and TARGET-AML. The data that support the findings of this study are available from the corresponding author upon reasonable request.

## Author Contributions

YW, JX, and YL: conceptualization. YW and FH: methodology and writing original draft. JL: validation. FH, YW, and RN: formal analysis and investigation. YL: resources, supervision, project administration, and funding acquisition. YC: data curation. YW, FH, and JL: writing, review, and editing. SC, LS, and DD: supervision.

## Conflict of Interest

The authors declare that the research was conducted in the absence of any commercial or financial relationships that could be construed as a potential conflict of interest.

## Supplementary Material

Supplemental Figure 1 Flowchart of research design. Metabolic risk score model was constructed using GSE37642 datasets based on the LASSO Cox regression and validated in the independent adult external cohort (TCGA dataset) and pediatric cohort (TARGET dataset).

Supplemental Figure 2 Kaplan–Meier curvers of the 9 metabolic gene expression in training cohort. **(A)** *ALDH2*, **(B)** *CYP2E1*, **(C)** *DNMT3B*, **(D)** *ENPP2*, **(E)** *HAAO*, **(F)** *ITPKA*, **(G)** *PAFAH1B2*, **(H)** *PHGDH*, and **(I)** *PSAT1*.

Supplemental Figure 3 Kaplan–Meier curve of the metabolic risk model in the younger population (≤ 65 years) of the training cohort **(A)** and adult external cohort **(B)**.

Supplemental Figure 4 Metabolic risk score of the paired AML samples at diagnosis and relapse in TARGET dataset.

Supplemental Table 1 Correlation between clinicopathological characteristics and metabolic risk level in training cohort, adult external cohort, and pediatric external cohort.
