# Supplementary figures and images for: Systematic Construction and Validation of a Metabolic Risk Model for Prognostic Prediction in Acute Myelogenous Leukemia
Source: Front Oncol. 2020 Apr 21;10:540. doi: 10.3389/fonc.2020.00540 (PMC7186449; doi:10.3389/fonc.2020.00540)

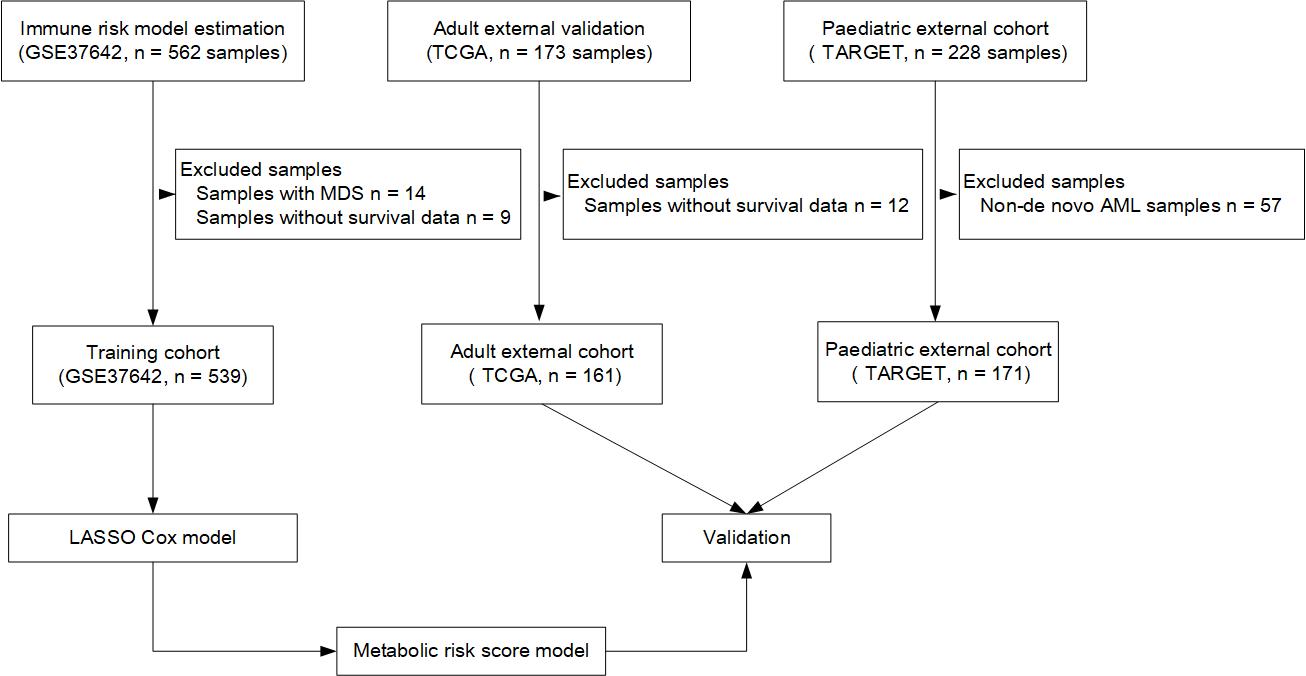

Supplement: Supplemental Figure 1 — Flowchart of research design. Metabolic risk score model was constructed using GSE37642 datasets based on the LASSO Cox regression and validated in the independent adult external cohort (TCGA dataset) and pediatric cohort (TARGET dataset). [file Image_1.JPEG]

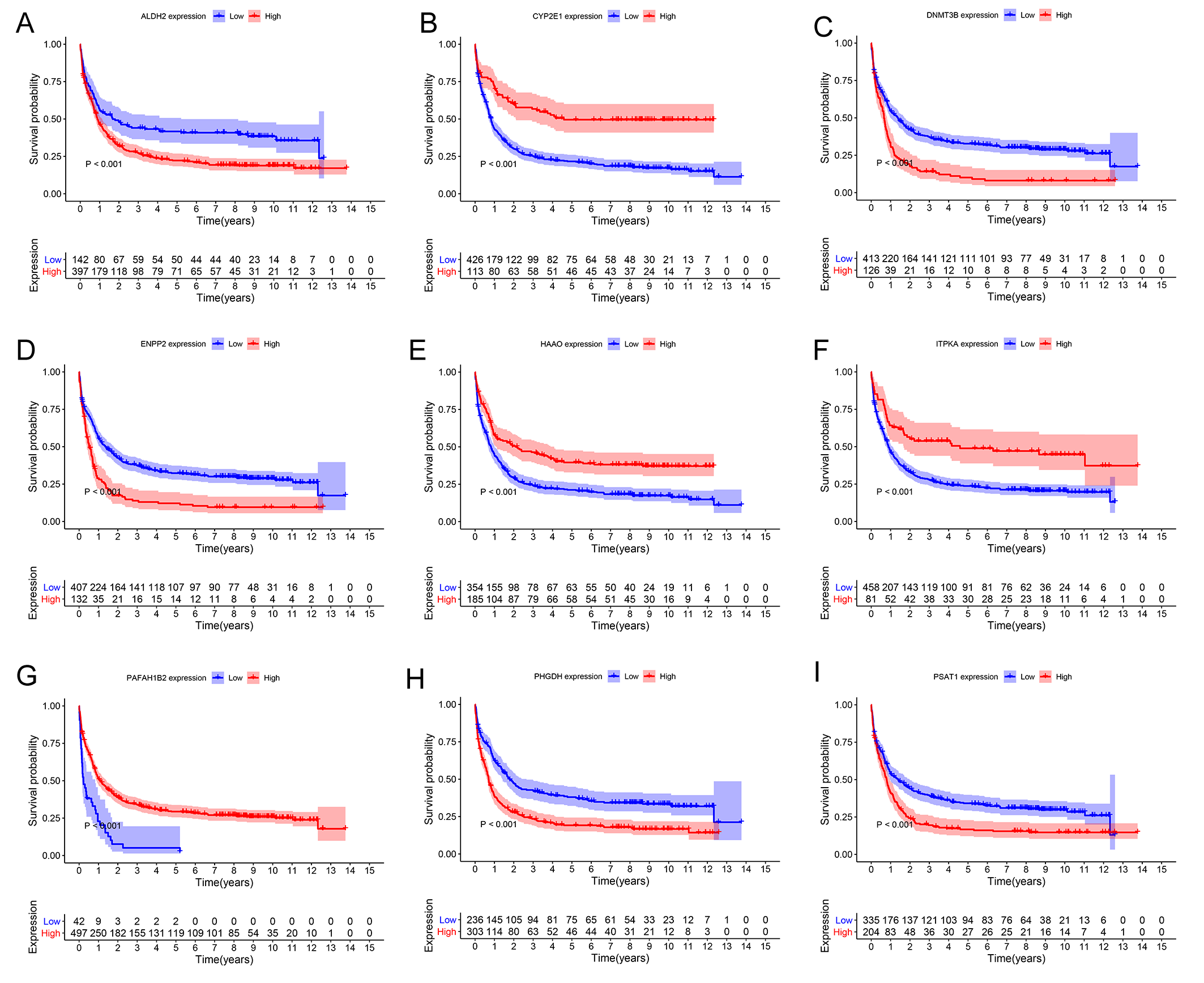

Supplement: Supplemental Figure 2 — Kaplan–Meier curvers of the 9 metabolic gene expression in training cohort. (A) ALDH2, (B) CYP2E1, (C) DNMT3B, (D) ENPP2, (E) HAAO, (F) ITPKA, (G) PAFAH1B2, (H) PHGDH, and (I) PSAT1. [file Image_2.TIF]

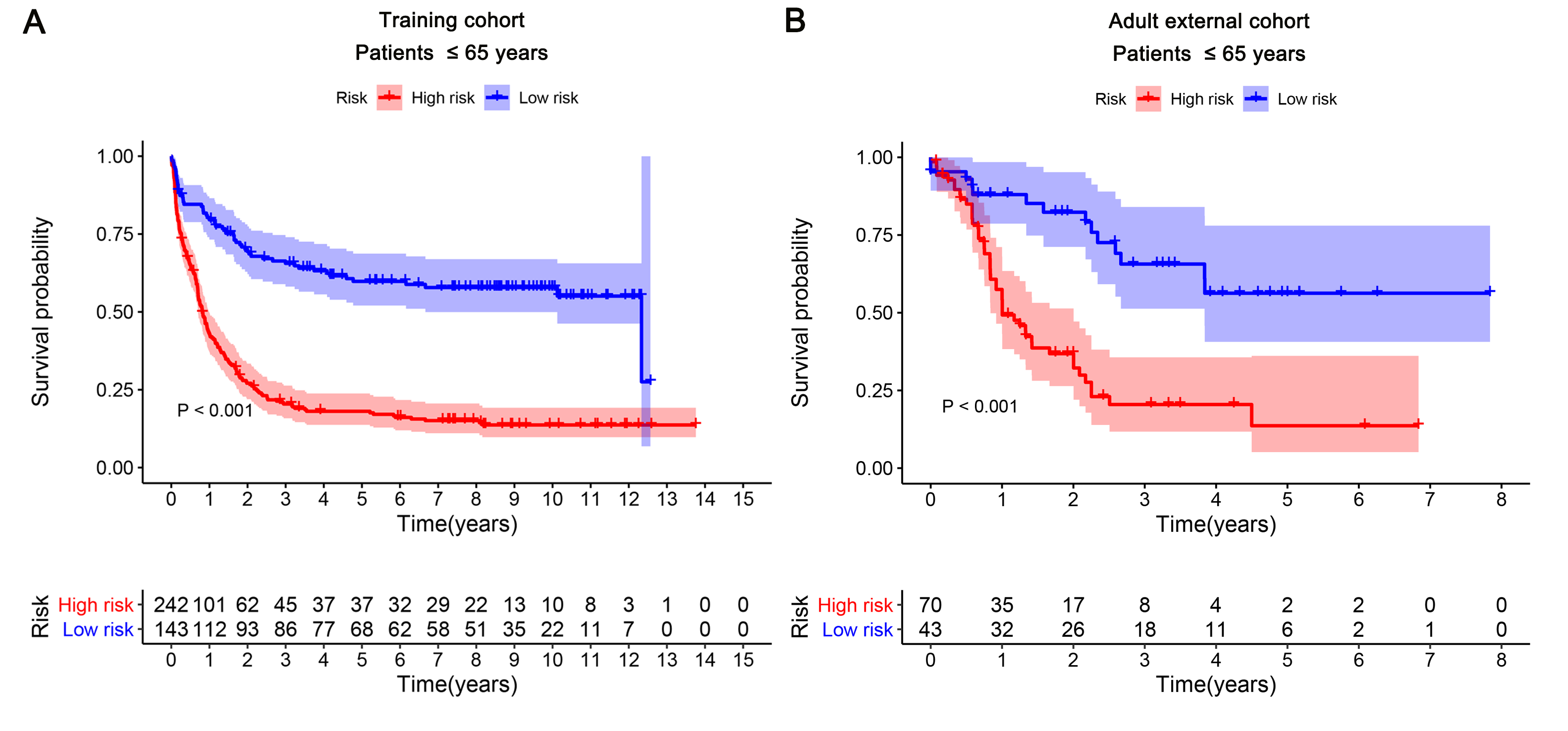

Supplement: Supplemental Figure 3 — Kaplan–Meier curve of the metabolic risk model in the younger population (≤ 65 years) of the training cohort (A) and adult external cohort (B). [file Image_3.TIF]

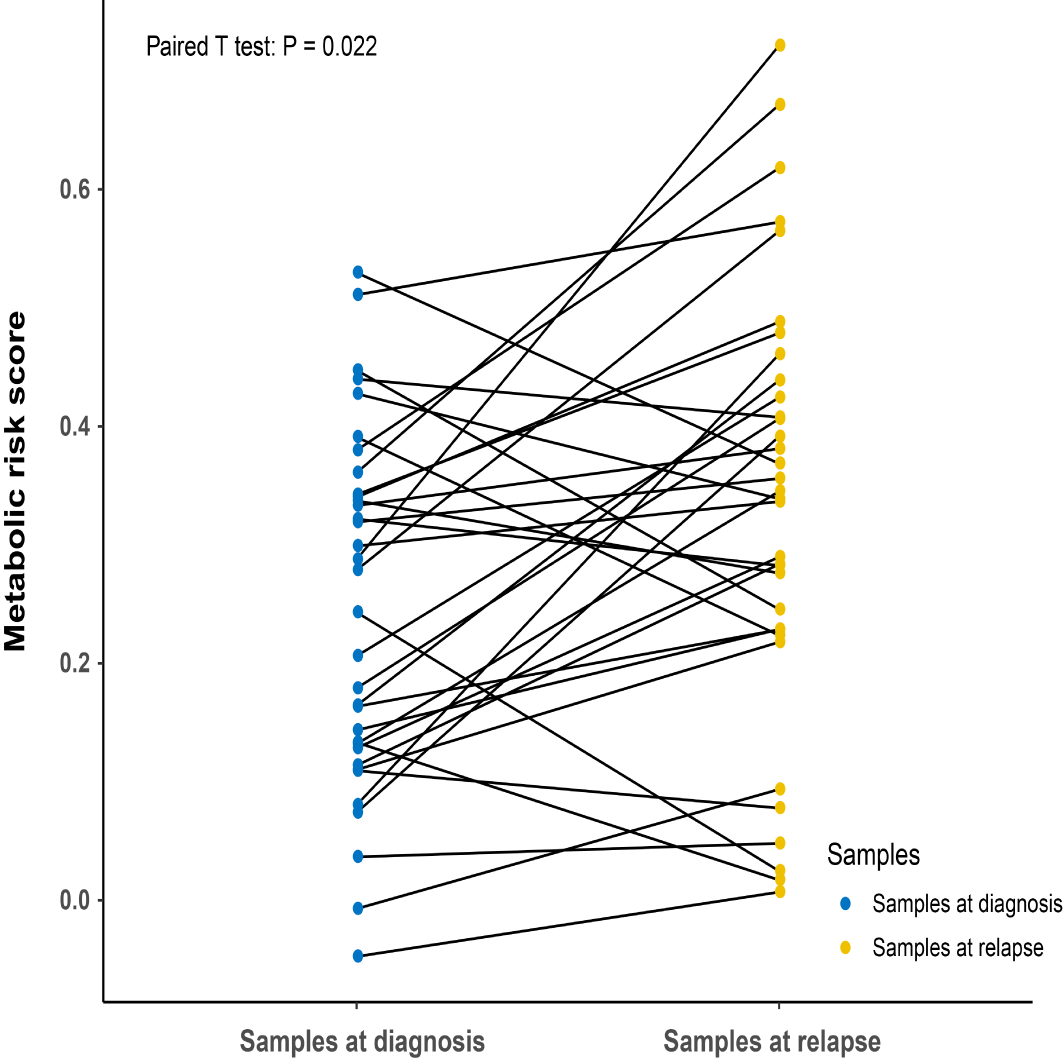

Supplement: Supplemental Figure 4 — Metabolic risk score of the paired AML samples at diagnosis and relapse in TARGET dataset. [file Image_4.TIF]
