# Supplementary material for: Systematic Construction and Validation of a Metabolic Risk Model for Prognostic Prediction in Acute Myelogenous Leukemia
Source: Front Oncol. 2020 Apr 21;10:540. doi: 10.3389/fonc.2020.00540 (PMC7186449; doi:10.3389/fonc.2020.00540)
Supplement: Supplemental Table 1 — Correlation between clinicopathological characteristics and metabolic risk level in training cohort, adult external cohort, and pediatric external cohort. [file Table_1.DOCX]

**Supplemental Table 1: Correlation of clinicopathological characteristics and metabolic risk level in training cohort, adult external cohort, and paediatric external cohort.**

|  |  | **Low risk (%)** | **High risk (%)** | **P-value** |
| --- | --- | --- | --- | --- |
| **Training cohort (GSE37642, N = 539)** | | |  |  |
| Age |  |  |  |  |
|  | ≤60 years | 127 (74.3) | 181 (49.2) | **< 0.001** |
|  | > 60 years | 44 (25.7) | 187 (50.8) |  |
| RUNX1-RUNX1T1 | |  |  |  |
|  | No | 145 (84.8) | 364 (98.9) | **< 0.001** |
|  | Yes | 26 (15.2) | 4 (1.1) |  |
| RUXN1 mutation^a^ | |  |  |  |
|  | Neg | 132 (92.3) | 287 (81.8) | **0.003** |
|  | Pos | 11 (7.7) | 64 (18.2) |  |
| FAB subtype^b^ | |  |  |  |
|  | M0 | 5 (2.9) | 17 (4.7) | **< 0.001** |
|  | M1 | 21 (12.4) | 92 (25.6) |  |
|  | M2 | 49 (28.8) | 111 (30.8) |  |
|  | M3 | 22 (12.9) | 4 (1.1) |  |
|  | M4 | 49 (28.8) | 71 (19.7) |  |
|  | M5 | 16 (9.4) | 49 (13.6) |  |
|  | M6 | 7 (4.1) | 14 (3.9) |  |
|  | M7 | 1 (0.6) | 2 (0.6) |  |
| **Adult external cohort (TCGA, N = 161)** | | |  |  |
| Age |  |  |  |  |
|  | ≤60 years | 37 (71.2) | 50 (45.9) | **0.003** |
|  | > 60 years | 15 (28.8) | 59 (54.1) |  |
| Gender | |  |  |  |
|  | Male | 27 (51.9) | 60 (55.0) | 0.710 |
|  | Female | 25 (48.1) | 49 (45.0) |  |
| Blast cells in BM (%) | |  |  |  |
|  | ≤ 70 | 37 (71.2) | 81 (74.3) | 0.672 |
|  | > 70 | 15 (28.8) | 28 (25.7) |  |
| Leukocyte count (x10^9/L)^c^ | | |  |  |
|  | ≤ 10 | 19 (37.3) | 45 (41.3) | 0.628 |
|  | > 10 | 32 (62.8) | 64 (58.7) |  |
| Hemoglobin (mg/dL)^c^ | |  |  |  |
|  | ≤ 80 | 12 (23.1) | 17 (15.7) | 0.259 |
|  | > 80 | 40 (76.9) | 91 (84.3) |  |
| Platelet count (x10^9/L) | |  |  |  |
|  | ≤ 40 | 29 (55.8) | 41 (37.6) | **0.030** |
|  | > 40 | 23 (44.2) | 68 (62.4) |  |
| Cytogenetic risk^d^ | |  |  | **< 0.001** |
|  | Faverable | 26 (50.0) | 4 (3.7) |  |
|  | Intermediate | 21 (40.4) | 73 (68.2) |  |
|  | Poor | 5 (9.6) | 30 (28.0) |  |
| FAB subtype | |  |  |  |
|  | M0 | 1 (1.9) | 14 (12.8) | **< 0.001** |
|  | M1 | 9 (17.3) | 25 (22.9) |  |
|  | M2 | 11 (21.2) | 26 (23.9) |  |
|  | M3 | 13 (25.0) | 1 (0.9) |  |
|  | M4 | 12 (23.1) | 22 (20.2) |  |
|  | M5 | 4 (7.7) | 17 (15.6) |  |
|  | M6 | 0 (0.0) | 2 (1.8) |  |
|  | M7 | 1 (1.9) | 2 (1.8) |  |
|  | Not Classified | 1 (1.9) | 0 (0.0) |  |
| **Paediatric external cohort (TARGET AML, N = 171)** | | | |  |
| Age |  |  |  |  |
|  | ≤ 11 years | 34 (54.8) | 66 (60.6) | 0.466 |
|  | > 11 years | 28 (45.2) | 43 (39.4) |  |
| Gender | |  |  |  |
|  | Male | 34 (54.8) | 53 (48.6) | 0.434 |
|  | Female | 28 (45.2) | 56 (51.4) |  |
| Blast cells in BM (%) | |  |  |  |
|  | ≤ 70 | 26 (43.3) | 41 (38.7) | 0.557 |
|  | > 70 | 34 (56.7) | 65 (61.3) |  |
| Leukocyte count (x10^9/L) | | |  |  |
|  | ≤ 10 | 5 (8.1) | 22 (20.2) | **0.037** |
|  | > 10 | 57 (91.9) | 87 (79.8) |  |
| CNS disease | |  |  |  |
|  | No | 58 (93.5) | 102 (93.6) | 0.994 |
|  | Yes | 4 (6.5) | 7 (6.4) |  |
| RUNX1-RUNX1T1 | |  |  |  |
|  | No | 49 (79.0) | 97 (89.0) | 0.076 |
|  | Yes | 13 (21.0) | 12 (11.0) |  |
| MLL rearrangements^e^ | |  |  |  |
|  | Neg | 55 (90.2) | 82 (82.8) | 0.199 |
|  | Pos | 6 (9.8) | 17 (17.2) |  |
| FLT3-ITD | |  |  |  |
|  | Neg | 60 (96.8） | 92 (84.4） | **0.013** |
|  | Pos | 2 (3.2) | 17 (15.6) |  |
| NPM mutaton^f^ | |  |  |  |
|  | Neg | 61 (98.4) | 98 (94.2) | 0.197 |
|  | Pos | 1 (1.6) | 6 (5.8) |  |
| WT1 mutation^g^ | |  |  |  |
|  | Neg | 60 (96.8) | 94 (89.5) | 0.091 |
|  | Pos | 2 (3.2) | 11 (10.5) |  |
| Cytogenetic risk^h^ | |  |  |  |
|  | Faverable | 46 (75.4) | 19 (18.6) | **< 0.001** |
|  | Intermediate | 15 （24.6） | 69 (67.6) |  |
|  | Poor | 0 (0) | 14 (13.7） |  |
| FAB subtype^e^ | |  |  |  |
|  | M0 | 1 (1.8) | 4 (4.0) | **0.006** |
|  | M1 | 6 (10.5) | 15 (14.9) |  |
|  | M2 | 14 (24.6) | 24 (22.8) |  |
|  | M4 | 24 (42.1) | 15 (14.9) |  |
|  | M5 | 7 (12.3) | 26 (24.8) |  |
|  | M6 | 0 (0.0) | 3 (3.0) |  |
|  | M7 | 1 (1.8) | 7 (6.9) |  |
|  | Not Classified | 4 (7.0) | 9 (8.9) |  |

a-h. missing data of 45, 9, 1, 2, 11, 5, 4 and 8 patients, respectively.
